# Reasons for disagreement regarding illnesses between older patients with multimorbidity and their GPs – a qualitative study

**DOI:** 10.1186/s12875-015-0286-x

**Published:** 2015-06-02

**Authors:** Heike Hansen, Nadine Pohontsch, Hendrik van den Bussche, Martin Scherer, Ingmar Schäfer

**Affiliations:** Department of Primary Medical Care, Center of Psychosocial Medicine, University Medical Center Hamburg-Eppendorf, Martinistrasse 52, 20246 Hamburg, Germany

**Keywords:** Disagreement, Chronic diseases, Focus groups, Illnesses, Multimorbidity, Physician report, Primary care, Qualitative study, Self-report

## Abstract

**Background:**

Chronic conditions are the most common themes in doctor-patient communication, especially for older patients with multimorbidity and their GPs. Former quantitative studies identified a variety of socio-demographic and health-related factors which were associated with the (dis-)agreement between medical records and patient self-reported diseases. The aim of this qualitative study was to identify reasons for disagreement regarding illnesses between patients and their GPs.

**Methods:**

We conducted three focus groups with GPs (n = 15) and three focus groups with multimorbid patients aged 65 to 85 (n = 21). The participants were recruited from the MultiCare Cohort Study. Focus groups were audiotaped and transcribed verbatim. The transcripts of the focus groups were analysed using the qualitative content analysis according to Mayring. Categories were determined deductively and inductively.

**Results:**

The analysis revealed seven themes concerning reasons for disagreement regarding illnesses between patients and their GPs: problems with communication and cooperation between health care professionals, disease management by the GP and the patient, the documentation behaviour of the GP, communication challenges between GP and patient, differences in the understanding of a disease between GP and patient, the prioritization and rating of diseases by GP and patient and obliviousness, repression and avoidance by the patient.

**Conclusions:**

For older patients with multimorbidity, our study demonstrated that there is a need to enhance the cooperation between GPs, specialists and outpatient care, a demand to improve doctor-patient communication and a need for interventions to increase patients’ knowledge of diseases.

**Electronic supplementary material:**

The online version of this article (doi:10.1186/s12875-015-0286-x) contains supplementary material, which is available to authorized users.

## Background

GPs are confronted with the phenomenon of multimorbidity more and more [1–3]. The management of older patients with multimorbidity is, in many ways, a challenge for the GPs. Sinnott et al. identified four problem areas in a synthesis of qualitative research: disorganization and fragmentation of healthcare, inadequacy of guidelines and evidence-based medicine, challenges in delivering patient-centred care and challenges in shared decision-making [4]. Successful communication between physicians, patients and other health care professionals is an important element for the management of patients with multimorbidity and a large part of the doctors’ duties [5, 6].

The management of chronic conditions is one of the most discussed themes in doctor-patient communication. In our MultiCare Cohort Study we analysed to what extent general practitioners (GPs) and their patients agree regarding the patients’ illnesses. 32 diagnosis groups were included in our analyses. A list of these diagnosis groups, the diagnoses’ prevalences and the positive agreement on these are shown in Table 1. The highest level of agreement was identified for the diseases diabetes mellitus and hypertension. Nine disease groups had a positive agreement between 80 and 61 % (e.g. chronic ischemic heart disease), eleven disease groups were between 60 and 41 % (e.g. cerebral ischemia/chronic stroke) and ten were between 40 and 10 % (e.g. gynecological problems). A variety of socio-demographic and health-related factors were associated with positive agreement, i.e. sex, age, education, income, disease count, depression, health-related quality of life (EQ visual analogue scale score) and nursing care dependency. For example: Women had a higher odds ratio for positive agreement with their GP regarding osteoporosis than men [7]. Other studies showed similar results [8–12].

**Table 1:** Prevalence and positive agreement of the 32 diagnosis groups: General practitioner reports vs. patient self-reports from the MultiCare Cohort Study (n = 3.189) [7]

Diagnosis group	Positive agreement^a^	Prevalence GP report % (n)	Prevalence patient self-report % (n)
Hypertension	0.89	77.9 (2,483)	72.3 (2,307)
Diabetes mellitus	0.87	37.6 (1,199)	31.1 (992)
Thyroid dysfunction	0.73	33.8 (1,077)	31.1 (992)
Parkinson’s disease	0.73	1.9 (62)	2.1 (67)
Asthma/COPD	0.70	24.2 (771)	22.0 (700)
Lipid metabolism disorders	0.69	58.5 (1,867)	45.8 (1,460)
Chronic ischemic heart disease	0.68	31.4 (1,000)	30.3 (966)
Chronic low back pain	0.67	49.5 (1,577)	62.2 (1,984)
Joint arthrosis	0.66	43.3 (1,382)	66.5 (2,120)
Osteoporosis	0.65	19.8 (632)	21.6 (690)
Cardiac arrhythmias	0.64	26.9 (858)	33.0 (1,053)
Cerebral ischemia/ Chronic stroke	0.60	11.8 (376)	13.9 (444)
Cancers	0.57	18.3 (584)	10.8 (343)
Lower limb varicosis	0.53	23.3 (742)	36.2 (1,155)
Prostatic hyperplasia (n = 1298)	0.50	27.9 (362)	39.4 (511)
Severe vision reduction	0.47	18.9 (604)	44.0 (1,403)
Hyperuricemia/Gout	0.44	17.3 (552)	16.8 (536)
Intestinal diverticulosis	0.44	14.5 (462)	13.6 (435)
Psoriasis	0.44	3.6 (116)	6.7 (213)
Atherosclerosis/PAOD	0.42	16.7 (531)	11.1 (354)
Renal insufficiency	0.42	10.7 (340)	9.7 (308)
Cardiac valve disorders	0.41	9.4 (300)	9.9 (317)
Chronic cholecystitis/ Gallstones	0.39	7.9 (251)	8.5 (272)
Cardiac insufficiency	0.36	13.1 (417)	17.2 (548)
Anemias	0.36	4.3 (136)	5.3 (170)
Neuropathies	0.35	14.7 (469)	35.6 (1,136)
Migraine/chronic headache	0.34	3.5 (113)	6.1 (196)
Rheumatoid arthritis/ Chronic polyarthritis	0.32	4.2 (134)	12.9 (411)
Urinary tract calculi	0.27	1.8 (58)	3.9 (124)
Dizziness	0.25	7.7 (246)	35.0 (1,115)
Hemorrhoids	0.24	7.5 (239)	22.8 (727)
Gynecological problems (n = 1891)	0.10	3.4 (64)	13.1 (248)

COPD: chronic obstructive pulmonary disease, PAOD: peripheral arterial occlusive disease

^a^Positive agreement (PA) is calculated with the formula: PA = 2a/(2a + b + c)

However, these studies might have missed some reasons, because no qualitative study was conducted before and not all reasons for disagreement might by accessible in quantitative designs. Therefore, the aim of this study was to perform focus groups with multimorbid patients and their GPs, in order to identify patient-related and provider-specific reasons for the disagreement regarding illnesses between patients and their GPs.

## Methods

### Study design

This qualitative study is based on three focus groups with GPs and three focus groups with primary care patients, which were conducted at the end of 2013. The participants were recruited from the MultiCare Cohort Study. This is a multicenter, prospective, observational cohort study with a total of 3,189 multimorbid patients in the age group 65 to 85, recruited from general practices in Germany. Multimorbidity was defined as the coexistence of at least three chronic conditions from a list of 29 diseases published elsewhere [13]. The methods used in this study, the patient population, and the sampling and response rates have been described in other papers [13, 14].

We chose focus groups as a qualitative method because we expected these yield comprehensive explanations for disagreement due to the group dynamics and the exchange of experiences by the participants.

For this qualitative study we asked the GPs of the MultiCare Cohort Study from the study centre Hamburg (n = 52) to participate in the focus groups. The GPs were invited by letter. Fifteen GPs were able to participate. Afterwards, we also invited their patients (also study participants of the MultiCare Cohort Study) by letter (n = 106) to participate in focus groups. We received 56 responses from potential participants, which were assigned to focus groups according to planned dates and group composition. We conducted three gender-specific patient groups: a male group (n = 7), a female group (n = 7) and a mixed gender group (n = 7). Our hypothesis was that some gender related disease topics could be discussed more openly when only men or women were present. 21 patients were able to participate.

### Characteristics of the study participants: Patients and GPs

The socio-demographic characteristics of the study’s participants (GPs and patients) are shown in Tables 2 and 3.

**Table 2:** Characteristics of the study participants: GPs

Pseudonym	Age	Gender	Years of practice	Number of patients treated in practice in each quarter	Number of physicians working in practice	Focus group No
GP1	52	male	14	500 thru 749 patients	1	1
GP2	60	male	22	500 thru 749 patients	1	1
GP3	57	male	14	1,000 and more patients	2	1
GP4	57	female	15	750 thru 999 patients	2	1
GP5	65	female	24	499 thru less patients	1	1
GP6	50	female	8	1,000 and more patients	1	1
GP7	52	female	12	500 thru 749 patients	3	1
GP8	61	male	28	750 thru 999 patients	3	2
GP9	42	male	9	750 thru 999 patients	2	2
GP10	55	female	16	750 thru 999 patients	2	2
GP11	47	female	8	1,000 and more patients	2	3
GP12	59	female	20	1,000 and more patients	3	3
GP13	59	female	14	500 thru 749 patients	1	3
GP14	50	female	7	500 thru 749 patients	3	3
GP15	39	female	7	500 thru 749 patients	4	3

**Table 3:** Characteristics of the study participants: Patients

Pseudonym	Age	Gender	Marital status	CASMIN Grade	Focus group No
P1	71	male	married	3	4
P2	80	male	married	not reported	4
P3	86	male	widowed	2	4
P4	72	male	married	2	4
P5	72	female	married	1	4
P6	76	female	married	1	4
P7	72	female	divorced	3	4
P8	88	male	married	1	5
P9	80	male	married	1	5
P10	72	male	married	1	5
P11	78	male	married	3	5
P12	80	male	widowed	3	5
P13	84	male	divorced	2	5
P14	70	male	married	3	5
P15	72	female	widowed	2	6
P16	72	female	married	1	6
P17	73	female	widowed	1	6
P18	82	female	married	3	6
P19	71	female	never married	2	6
P20	87	female	widowed	2	6
P21	78	female	widowed	1	6

The participating GPs were between 39 and 65 years old (mean age 53.4). Five of the 15 GPs were male. The years of practice experience ranged between 7 and 28 years (mean 14.6). Most of the GPs (42.9 %) treated between 500 and 749 patients each quarter (3 month period) and worked in single practices (35.7 %).

The patients were between 70 and 88 years old (mean age 77.0). 10 of the 21 participants were female. 12 participants were married, two divorced, six widowed and one never married. Seven patients had a low education level (CASMIN grade 1, [15]), another seven had a medium education level (CASMIN grade 2) and six had a high education level (CASMIN grade 3).

### Interview guide and data collection

For the qualitative study presented here, we developed an interview guide to explore the disagreement between patients’ self-report about chronic conditions and doctors’ diagnosis, which were identified in the preliminary study by Hansen et al. [7].

The interview guide for GPs started with general questions about communication problems between GPs and their patients e.g. “When you think of conversations with your multimorbid, elderly patients, are there scenarios where you had the feeling that your patient did not understand everything? Or that the patient couldn’t or didn’t want to listen to you?” Thereafter, we presented the results from the analysis of the agreement between self-reported and general practitioner-reported chronic conditions among the participants of the MultiCare Cohort Study and asked the GPs about their impressions as well as which reasons they could imagine being responsible for any disagreements. The interview guide for patients had a similar structure. The questions, as well as the presentation of the results, were adapted for patients. To support the interaction in the focus groups we kept important keywords on a pin board. The interview guides are shown in the Additional files 1 and 2.

The focus groups were led by HH and IS, lasted approximately 120 min and were recorded on audiotape. HH is a health scientist and IS a sociologist; both are involved in health services research and epidemiological research on multimorbidity.

### Coding and analysis

Audiotapes were transcribed verbatim by trained research assistants and, afterwards, were checked and corrected where necessary by HH.

The transcripts were analysed using the qualitative content analysis according to Mayring [16]. This reductionistic method condenses the large amounts of data to identify the main content. Based on the literature, on the interview guide’s topics and on the keywords on the pin board, we developed deductive categories for coding. For example: The category “Communication challenges between GP and patient” included the code “too little time for consultation”. The coded text segments varied from short sentences to long paragraphs. The coding was performed using MaxQda11 [17]. HH and IS read and coded the transcripts independently and discussed the categories afterwards. During the coding process, we added inductive categories as they arose from the material. Afterwards HH and IS conclusively condensed the categories and summarized them into seven themes. The final set of categories and themes were determined by consensus.

### Ethical considerations

The study was approved by the Ethics Committee of the Medical Association of Hamburg (approval no. 2881) on 8.11.2013. All participants gave a written, informed consent to participate in the study.

## Results

We identified seven themes concerning reasons for disagreement regarding illnesses between patients and GPs, namely: problems with communication and cooperation between health care professionals, disease management by the GP and the patient, the documentation behaviour of the GP, communication challenges between GP and patient, differences in the understanding of a disease between GP and patient, the prioritization and rating of diseases by GP and patient and obliviousness, repression and avoidance by the patient. Most categories were reported by both perspectives: GP and patient. Themes, categories and perspectives are summarized in Table 4.

**Table 4:** Themes and categories of reasons for disagreement regarding illnesses between older patients with multimorbidity and their GPs

No	Theme	Perspective	
	Category	GP	Patient
**1**	Problems with communication and cooperation between health care professionals		
	The hospital communicates many diagnoses that the general practitioner considered incorrect or exaggerated	×	×
	The specialist explains his findings too little and / or responds insufficiently towards the patient	×	×
	The GP is not/ or inadequately informed by the specialists, e.g. because no medical report is transferred	×	×
	The GP is not involved in the treatment by the specialist	×	×
	The patient reluctantly reports being treated by an alternative practitioner	×	
**2**	Disease management by GP and patient		
	The GP’s and patient’s understanding of a disease agree more in diagnoses requiring regular disease management	×	×
	The GP’s diagnostic process is more difficult, if the examination of the patient is uncomfortable for the GP or he/she does not feel responsible	×	×
	The complexity of a single or multiple diseases complicates the GP’s diagnostic process and the clinical management, as seen for example in multimorbidity	×	×
	In patients who take own initiatives, there is a greater consistency in disease understanding between GP and patient		×
**3**	Documentation behaviour of the GP		
	The pressure from health insurances to encode certain diseases, affects the documentation behaviour of the GP	×	
	Whether a disease is diagnosed by the GP or not, depends on the disease stage and measured values	×	×
	Not all symptoms are documented by the GP as diseases	×	×
	The GP has little knowledge about diseases that are lie far in the patients’ pasts	×	×
	Errors in the patient record cannot be corrected subsequently, e.g. in the hospitals’ or GPs’ records	×	×
**4**	Communication challenges between GP and patient		
	There is too little time to discuss complaints and diseases to achieve a mutual understanding	×	×
	The GPs’ medical understanding of illnesses must be translated into the patients’ level of understanding and vice versa	×	×
	The consultation can be exhausting and this may cause something to be forgotten or missed	×	
	The exchange of information and disease management are dependent on the doctor-patient relationship and the mutual trust	×	×
	Agreement on understanding a disease is worse in patients who are difficult to lead and / or functionally impaired	×	
**5**	Differences in the understanding of a disease between GP and patient		
	Information that is given by specialists and / or elaborate diagnostics are formative for the patient	×	
	The patients’ clinical pictures are influenced by the media and campaigns	×	×
	The GPs’ medical understanding of an illness deviates from the patients’ everyday understanding	×	×
**6**	Prioritization and rating of diseases by GP and patient		
	The GP prioritizes diseases that affect the prognosis of the patient	×	
	The patient prioritizes diseases associated with complaints	×	
	Diseases that are not relevant for the patient from a general practitioner's point of view are not communicated to the patient	×	
	Diseases that are not relevant for the GP, from a patient’s perspective, are not communicated to the GP	×	×
**7**	Obliviousness, repression and avoidance by the patient		
	The patient does not remember diseases if they are too far in the past	×	×
	Diseases are repressed by the patient, e.g. cancer	×	×
	The patient conceals embarrassing diseases	×	×
	The patient tries to avoid the utilization of health services	×	×

**× =** Category has been reported by the respective perspective (GP, patient)

### Theme 1: Problems with communication and cooperation between health care professionals

Both GPs and patients reported cooperation and communication problems with other health services. Missing or incorrect information exchange between hospitals, specialists, other health services and GPs made the doctor-patient communication difficult and influenced the patients’ disease awareness.

**The hospital communicates many diagnoses that the general practitioner considered incorrect or exaggerated.** GPs received incorrect diagnoses from hospital reports, particularly secondary diagnoses were inflated possibly due to financial incentives for overdiagnosing in the German DRG-system (diagnosis related groups) or inaccurate quality management in the hospitals. *“Dehydrated patients are admitted to hospital, they have a bad creatinine [level], and then they have a renal insufficiency [according to the coding of the hospital] and then it shows up in a report and then it is copied from one report to the other, and the text block remains unchanged at the hospital and is added again at the next hospital stay and is not becoming any more real…” (GP12)*

**The specialist explains his findings too little and/or responds insufficiently towards the patient.** Patients and GPs reported that the specialists often spoke little to not at all about the results of their examinations. Time constraint in specialists’ practices was mentioned as a reason for this phenomenon. Therefore, the patients had to go to their GP to discuss the results. “*Again and again patients [turn] up […] telling us that they would like us to explain their diagnoses because they are not explained thoroughly elsewhere […]. But I am also quite sure that some [patients] do not do that. They still don’t know what they are suffering from afterwards but they don’t ask us either.” (GP11)*

**The GP is not/ or inadequately informed by the specialists, e.g. because no medical report is transferred.** Some specialists rarely, if at all, wrote medical reports and sent them to the GPs. Patients procured their medical reports. Sometimes specialists refused the GP’s referral from the patients to avoid the then mandatory reporting. *“Also, patients tell me that specialist colleagues sometimes do not want to have their referral.” (GP12) “Certainly, because they are required to mandatory reporting, that’s for sure.” (GP13) “Which means […] they are [not] accepting the […] referral. Certainly, this is […] a problem.” (GP12)*

**The GP is not involved in the treatment by the specialist.** If patients contacted a specialist without former appointment with the GP and without a referral, the specialists would not inform the GP. *“[In] orthopaedics […] [it] works particularly poorly […] This is where they […] enter into therapy contracts […] with patients, which […] circumvent or run parallel to the general practitioner’s, of which there is no documentation at all because they often are services which have to be paid for privately.” (GP13)*

**The patient reluctantly reports being treated by an alternative practitioner.** GPs rated the treatment by an alternative practitioner as a sensitive topic because it is a separate treatment with a competitive character. *“They get diagnoses from natural health professionals but they don’t tell you anything about it […]. This is often a parallel world we don’t know anything about […]. You are surprised sometimes what they have standing around at home.” (GP13)*

### Theme 2: Disease management by GP and patient

There was a variety of factors related to disease management, which could influence the (dis-)agreement regarding illnesses between GPs and patients.

**The GP’s and patient’s understanding of a disease agree more in diagnoses requiring regular disease management.** Diseases from disease management programs (DMPs), diseases bearing a high risk of death, measurable diseases e.g. through laboratory parameters, and diagnoses requiring many medications and/or a lot of regular consultations were subject to a better agreement between GPs’ and patients’ understanding of disease. *“The high rate of conformities are indeed the diseases which bind the patients to us quite regularly. No matter whether DMP or […] thyroid diseases, they come for a regular metabolic monitoring, which is […] taken incredibly serious […] by the patients.” (GP7)*

**The GP’s diagnostic process is more difficult if the examination of the patient is uncomfortable for the GP or he/she does not feel responsible.** Patients and GPs mentioned medical areas for which the family doctor seemed to not be responsible or which the family doctor did not consider pleasant. *“[I] believe physicians are not as keen on performing a rectal examination as on just quickly* […] *auscultating the heart.” (GP3) “Haemorrhoids are last on the list, this is something [the physician] is also not really fond of looking at.“(P11)*

**The complexity of a single or multiple diseases complicates the GP’s diagnostic process and the clinical management, as seen, for example, in multimorbidity.** Patients told tales of very long diagnostic processes, often with detours. GPs emphasized the complexity of the disease management in patients with multimorbidity. *“The really multimorbid patient, well, the one with a multitude of diagnoses, this is where something easily slips through your fingers so that you don’t examine and treat everything […] with the same attention. Well now, this is surely more often the case with me, that I overlook something altogether.” (GP14)*

**In patients who take own initiatives, there is a greater consistency in disease understanding between GP and patient.** This reason for better agreement was only revealed in the patient focus groups. If patients demanded treatment and actively asked the GP and other doctors or informed themselves, the understanding of the illness would overlap more and the disease management would be more successful.*”I’ve learnt from experience that, if I do not write everything on a large sheet of paper, and do not go over it exactly point by point, something will often go amiss at the general practitioner’s. Well, he asks a lot and does a lot, and for me he is an incredibly fantastic general practitioner, but you have to be a little bit on the lookout.” (P10)*

### Theme 3: Documentation behaviour of the GP

The documentation behaviour of the GP was an important factor regarding the concordance between GP and patient. It was influenced by several different aspects.

**The pressure from health insurances to encode certain diseases, affects the documentation behaviour of the GP.** GPs reported that health insurances occasionally made proposals for encoding certain diseases, in order to get more funds from the morbidity-oriented risk adjustment scheme within the statutory health insurance system in Germany. For this reason a lot of ICD codes were assigned by GPs which were not in line with patients’ awareness of their diseases.*”We have to say loud and clear that we have also been under a lot of pressure by part of our self-management since […] two, three years, that we are supposed to encode a lot, because then there is more money.” (GP13)*

**Whether a disease is diagnosed by the GP or not, depends on the disease stage and measured values.** The GPs assigned a diagnosis due to very low increases in certain values (e.g. renal insufficiency by slightly increased creatinine values) in order to monitor these but did not always tell the patient about the diagnosis. *“These stages 1 and 2 most certainly [fall] under renal insufficiency […], those who have a crea below 2, that is something that the patient does not […] consider to be […] renal insufficiency, he/she would say: “Yes, the kidney values are not completely normal.” However, he/she doesn’t consider this to be a disease.” (GP8)*

**Not all symptoms are documented by the GP as diseases.** Some symptoms were not classified as diseases by the GP, e.g. dizziness, especially when the symptoms were not life-threatening. For the patients these symptoms could have been very relevant. *“And only those are diagnosed with vertigo who are really severely afflicted with it and really complain a lot […]. The rest I simply brush off.” (GP11)*

**The GP has little knowledge about diseases that lie far in the patients’ pasts.** Very old diseases were not documented in the GPs’ medical records and the collecting of medical results was rated as very tedious by the GPs. *“Regarding the […] kidney stones I can certainly imagine that someone once had a renal colic and that he/she remembers it for the rest of his/her life, and it happened 20 years ago, which you, as general practitioner, have already forgotten.” (GP2)*

**Errors in the patient records cannot be corrected subsequently, e.g. in the hospitals’ or GPs’ records.** This reason was reported by GPs and patients. One patient reported that a mistake, made in his medical records during a hospital stay, could not be corrected anymore and he criticized the credulity of written medical reports: *“Some scatterbrain once wrote in my bulky medical records, which […] comprise several folders, that I had two stents and […] a bypass. […] And, to boot, there are physicians who, unfortunately, are reading it thoroughly and tell you afterwards: “Now, we will have to check the condition of your stents.” And when I reply: “I haven’t got any.” They say: “But that’s what’s written in your medical record report.” (P1)*

### Theme 4: Communication challenges between GP and patient

We found five categories on the subject communication challenges. Main problems were: not enough time for conversations, a different understanding of illness, complicated or functionally impaired patients and a lack of mutual trust.

**There is too little time to discuss complaints and diseases to achieve a mutual understanding.** GPs explained that they need a lot of time to talk about the complaints and/or diseases with their patients until they feel that their patients are well informed. Sometimes GPs needed a lot of time to understand the patients. In any case, there was not enough time for detailed conversations due to full waiting rooms. Therefore, patients reported that their GP was too fast. Some patients did not take the time because they thought the GP had no time. GPs mentioned that they had no time to ask. *“Inquiring […] is one thing, the other is this saying: “Whoever asks gets answers.” And the time […] to have the patient come in once again […], this is when I always notice that I do not want that at all. We have worked through 2 or 3 issues, the waiting area is jam-packed and, actually, I really don’t have [time] anymore, or I am glad when the patient is […] leaving and the next one comes in […] Although I know that I might do him injustice or there is still something, I am really not prepared to do so any longer.” (GP3)*

**The GPs’ medical understanding of illnesses must be translated into the patients’ level of understanding and vice versa.** GPs rated the mediator function as one of their most common functions. Patients mentioned that they did not always understand the physicians’ technical terms and / or that they had the feeling that the GP was concealing some information.*” The question of what the patient understands to be a certain disease and what I understand to be a certain disease. And what consequences should follow from these things […] This middleman function […] between the scientific medical concepts and the every-day communication experience. This is what we do very, very often.” (GP8)*

**The consultation can be exhausting and this may cause something to be forgotten or missed.** GPs saw the consultation as a strenuous conversation for the patients. Patients could not register everything told to them by their physician at once. And patients did not always directly mention everything. Many concerns would be mentioned at the end of the consultation. Some patients were very demanding so that the GP missed something. *“And then, of course, there are these annoying patients where you think: “Oh no, not again, she always gets here at quarter past 12 and settles down comfortably and then I open the conversation with the question: “What can I do for you today?” and not with: “How are you?” because it is a question I dare not risk to ask at all […] because it would mean an extra half an hour whereas I simply try to restrain patients […] and thus it certainly also happens that I […] miss and overlook things.” (GP11)*

**The exchange of information and disease management are dependent on the doctor-patient relationship and the mutual trust.** A long relationship between the GP and the patient encouraged disease management. The agreement between the GP and the patient was estimated to be better when the patients had a GP that suited them and with whom they felt satisfied. *“I suppose you have to trust. Well, I feel whether he does me good, that he responds to my problem, anyway, that I am not just a number but that he sees me as a person.” (P17)*

**Agreement on understanding a disease is worse in patients who are difficult to lead and/or functionally impaired.** GPs had the feeling that patients’ concepts of illness were often resistant to change and noticed a lack of insight by patients regarding their illnesses. They also reported that patients often do not communicate openly and that functional impairments, e.g. dementia or hearing loss made communication more difficult.*” The hard of hearing […] nod [often] and say yes […]. That they do not want to admit that they don’t understand everything, and consequently a lot of information is lost, and even if you already know it, you are speaking slowly, but sometimes you forget to do so when you are in a hurry, and I believe that they really have a higher risk because the communication is muddled.” (GP11)*

### Theme 5: Differences in the understanding of a disease between GP and patient

We identified a few reasons which caused differences in the understanding of diseases between GPs and their patients. Patients were influenced by information from specialists, media or campaigns and hold onto their understanding of the illness.

**Information that is given by specialists and / or elaborate diagnostics are formative for the patient.** GPs reported that patients were very susceptible to diagnoses which are justified by extensive diagnostics and to exaggerated instructions by specialists. This could lead to misunderstandings. *“It is often the case that patients […] say: “I’ve had a stroke,” and when I look at their file, I simply cannot find this stroke anywhere. In the end, it turns out that the patient had been admitted to hospital with certain symptoms at some time or another […] and they said: “Yes, it might have been a TIA,” however, this was passed on to the patient at the hospital as: “You had a minor stroke”. He is sent back home after three days supplied with ASS and I am looking at the whole thing and say […] you can look at it this way, if you like, but you don’t have to look at it […] this way.” (GP8)*

**The patients’ clinical pictures are influenced by the media and campaigns.** Patients informed themselves through the press, television or internet. Fashionable diseases were often remembered. *“An incredibly triggered interest emerged in the population whether they’ve got osteoporosis or not. […] An unbelievable hype was created with this vitamin D and what not. Indeed I believe that, for this reason, everyone [will] […] inquire whether he/she has osteoporosis, and everyone is already inquiring after their cholesterol, no matter how healthy they are, marathon runners and non-smokers and what not, but they all want to know their cholesterol level.” (GP7)*

**The GPs’ medical understanding of an illness deviates from the patients’ everyday understanding.** Most patients had an everyday understanding of illnesses which can cause misunderstandings. Patients had colloquial definitions of diseases e.g. rheumatoid arthritis vs. joint arthrosis. GPs reported that patients often had no understanding for the chronicity of an illness, which hindered the treatment of chronic diseases such as high blood pressure. GPs also recounted that patients thought that the disease had to be found where the symptoms were located: *“[The patient] suffered […] a new occurrence of atrial fibrillation with quite severe shortness of breath, he was extremely short-winded and I wanted to tell him that he should see a cardiologist now because I wanted to rhythmise him or I simply wanted to admit him to hospital, he was simply doing too poorly. […] “What am I supposed to do at the cardiology clinic,” he absolutely could not understand why and to explain it to him now that his heart was practically causing a congestion of fluids in his lungs and that his heart was the real cause of it, while he insisted again and again to see his pulmonologist […] He was very enthusiastic afterwards because he was [had been] admitted to the cardiology clinic.” (GP10)*

### Theme 6: Prioritization and rating of diseases by GP and patient

Patients and GPs reported a different prioritization of diseases. In some cases GPs and patients did not inform each other about diseases which were, from their point of view, unimportant.

**The GP prioritizes diseases that affect the prognosis of the patient.** GPs reported that patients seemed unaware of some diseases which were important to the GP e.g. renal insufficiency, anaemia, hyperuricemia, pre-diabetes, high blood pressure (asymptomatic) or lipid metabolism disorders.*” There are diagnoses which are objectively dangerous for patients because they shorten their life expectancy, and it is exactly this we usually tinker with and put our efforts in. And the others are diagnoses which have negative effects on the patients’ quality of life, they are, however, also important issues, and it is precisely those which are often dealt with inadequately. I mean urinary and stool incontinence, […], which don’t shorten lives but…” (GP4)* GPs rated diseases that cause only discomfort, but do not affect the prognosis, as not very important. *“And it is the issues of mental wellbeing, back ache, migraine where we could also say, well, if we had a huge amount of time, we could take care of all health issues, but we haven’t got any and therefore have to focus a bit.” (GP13)*

**The patient prioritizes diseases associated with complaints.** GPs suspected that their patients were more aware of diseases with dominant complaints and rated them as more important, whereas GPs could forget them: *“It is precisely […] this vertigo, at the moment I have one too, […] I talk [to her] for a quarter of an hour about this and that every time after which she replies, “but my vertigo,” and I answer every time, well, unfortunately there is nothing I can do about it, we have already tried and done everything. But it is probably the first diagnosis she will mention: “What are you suffering from?”. “Vertigo”. For me, this would be somewhere all the way at the bottom, it might well be that I block it out as well.” (GP15)*

**Diseases that are not relevant for the patient from a general practitioner's point of view are not communicated to the patient.** The GPs mentioned that they concealed diseases without consequences for the treatment or diseases that would only worry the patient e.g. low grade renal insufficiency or hyperuricemia. These diseases appeared only in the medical records. *“I suppose valvular heart disease is a documentation problem. She had been for a echocardiography once, somehow a first to second degree or first degree insufficiency occurs and is added to the diagnoses […] This doesn’t have to be of interest for the patient either.” (GP13)*

**Diseases that are not relevant for the GP, from a patient's perspective, are not communicated to the GP.** Complaints, that the patient treated himself or that went away by themselves, were not told to the GP. *“Sometimes, you have to be your own physician. Well, I don’t go there for every poppycock, when I have a headache or so.” (P19)*

### Theme 7: Obliviousness, repression and avoidance by the patient

There were several different reasons why patients forgot, repressed or avoided diseases, which were documented in the GP’s records.

**The patient does not remember diseases if they are too far in the past.** Patients forgot about old diseases documented in their medical records. *“I think the reason might also be that the diseases or symptoms occurred quite some time ago, so that you yourself are not thinking of them anymore, but as the general practitioner has got it all on his file.” (P5)*

**Diseases are repressed by the patient, e.g. cancer.** GPs reported that some patients hid complaints because they were afraid of malignant conditions or the patients did not admit certain diseases. *“The classic: “Diabetes? I certainly haven’t got diabetes.” This doesn’t happen daily, but [the] diagnosis is documented by blood sugar levels. […] “Well, you once mentioned something like minor adult-onset diabetes, but diabetes? Me?” This is one example, or when you find out somehow through twisted channels that they have discontinued the medication after all.” (GP11)*

**The patient conceals embarassing diseases.** Some patients reported that talking about embarrassing diseases with their GP was difficult. Others were afraid of diagnosis, relapses, side effects or surgery. *“I could imagine, particularly regarding haemorrhoids, this is certainly the kind of subject, well, you don’t talk about it with anybody at all, and not even with your general practitioner. Well, I suffered from it for a long time until I went to have surgery. But you know, these are topics you really don’t like to approach.” (P13)*

**The patient tries to avoid the utilization of health services.** GPs suggested that some patients were afraid of family members not being cared for if, for example, they had to go to the hospital. Some patients avoided referrals to specialists, which is why they hid their complaints. *“A female physician who thought, after a 24-h test, that my blood pressure was too high and I therefore had to take medication. “Oh,” I say, “always?” “Yes, always.” Well, and now we have postponed it for the time being, this was in the spring, but now I don’t dare to return there at all […]. Because I believe, it changes with the […] blood pressure. And next, the other thing would be […] I should have a colonoscopy. […] I know that something might happen during it and therefore I am still also a little bit apprehensive about it.” (P12)*

## Discussion

In addition to our previous research, we tried to identify further reasons for a lack of agreement between the understanding of illnesses in GPs and their patients. The focus groups with multimorbid patients and their GPs yielded seven themes of reasons for disagreement regarding illnesses between patients and their GPs . These themes concern the need to enhance the communication and cooperation between health care professionals, to improve the communication between GP and patient, and to increase the patients’ disease knowledge.

### Enhancing the communication and cooperation between health care professionals

There are several challenges in the management of chronic diseases, especially multimorbidity [4]. In our study, GPs and patients often reported interface problems between professionals and a lack of interdisciplinary communication. This is in line with the findings of other studies. Fried et al. identified barriers in clinicians’ preferred approaches to decision making in focus groups with physicians, nurse practitioners and physician assistants. One barrier was the interaction with specialists [18]. Smith et al. reported inter-professional communication difficulties as well [19]. A study from Gill et al. emphasized the uncoordinated health services from the patients’ points of view. Patients were frustrated over poor communication and a lack of care coordination [20]. Moßhammer et al. assessed relevant deficiencies in, and barriers to, the cooperation between general practitioners and occupational health physicians in Germany. They identified problems areas such as a lack of communication, insufficient cooperation and a lack of knowledge, prejudices, competition and mistrust between the two groups [21]. These studies showed that there is a great need for optimizing interface problems and interdisciplinary communication.

These problems could be handled by strengthening the role of the GP as a coordinator in the German health care system. This possibility has already been installed in a special GP-centred, health care contract (“Hausarztzentrierte Versorgung”, HZV) [22], which the German statutory health care funds are legally required to offer. The evaluation of these contracts by Klingenberg et al. found that 24.9 % of the surveyed GPs reported an improvement concerning the cooperation with specialists [22]. The HZV might, therefore, be a promising approach to improve interface problems and the lack of interdisciplinary communication. Nevertheless, there is a need for optimizing the reporting system in ambulatory care in Germany. The specialists’ obligation to submit a report to the GP should, therefore, be analyzed by independent studies and monitored by the National Association of Statutory Health Insurance Physicians in Germany.

### Improving the communication between GP and patient

A predominant problem, concerning the communication between doctors and patients, was the insufficient consultation time, which was also described in other studies [18, 19, 23–25]. In an international comparison, the median patient contact time was the lowest for German primary care practices with 9.1 min compared to a median ranging from 10.3 (Italy) to 28.8 (Sweden) minutes in the other countries [26]. Especially German patients with many chronic conditions might have too little time with their doctors to talk about their diseases and their treatment plan.

In our study we also identified different factors complicating communication, e.g. if patients were functionally impaired. Luijks et al. reported mental health problems as a major barrier negatively influencing the management of multimorbidity [24]. The synthesis of Sinnott et al. summarized cognitive impairment, poor social supports and finances, and a low level of motivation as factors influencing patients’ ability to understand and adhere to treatment [4]. On the other hand, a good, personal patient-doctor relationship positively influenced the communication and the management of multimorbidity [19, 24, 27].

Another challenge for the communication between GP and patient was the different prioritization of diseases. Our results suggested that GPs more often prioritized diseases that affect the prognosis, while patients more often prioritized diseases with impairments. In concordance with our results, Smith et al. described different goals of clinicians and their patients [19]. Another qualitative study from our MultiCare study group, by Löffler et al., focused on the dealing with multimorbidity. They conducted separate narrative interviews with GPs and their patients and found that patients and GPs had different priorities: GPs focused on the management of life-threatening diseases, while patients prioritized autonomy and social life [28]. A quantitative study by Zulman et al. demonstrated patient-provider concordance. Patients and providers where asked to rank their most important health concerns. Patients prioritized symptomatic conditions such as pain, depression or breathing problems more than their providers [29]. Other studies also showed differences between older patients and their general practitioners regarding prioritization [30–32].

To improve the communication between GP and patient regarding the management of multiple diseases, a common prioritization of the treatment goals could be useful. For example, this could be achieved by a narrative doctor-patient dialogue, which is currently being tested in a cluster-randomized controlled trial in Germany [33].

### Increasing the patients’ disease knowledge

Our focus groups pointed out that the patients’ diseases concepts were different from the doctors’ medical concepts. For example, patients did not distinguish between similar diseases e.g. rheumatoid arthritis and joint arthrosis. Or they had no understanding for the chronicity of an illness. This different understanding of diseases could cause misunderstandings that complicate the treatment, especially in multimorbidity.

Kivelä et al. demonstrated, in a systematic review, that health coaching had positive effects on patients’ physiological, behavioural and psychological conditions [34]. Therefore, patient education could improve patients’ disease knowledge and, thus, the compliance regarding the management of multiple chronic conditions. GPs should motivate their patients to participate in existing education programs for patients e.g. diabetes education [35]. Certainly, these education programs are limited in the statutory health insurance system in Germany. For this reason, the GPs role of explaining the diseases to their patients remains a great part of their work. In the development of clinical practice guidelines for multimorbid patients, the topic “patient education” should also be taken into account.

### Strengths and weaknesses

To our knowledge, this is the first study analysing reasons for disagreement regarding illnesses between patients and their GPs by performing focus groups with GPs and, in particular, with their multimorbid, older patients in Germany. We took into account both the providers’ and the consumers’ perspectives. The qualitative approach added further reasons for a lack of agreement between the GP and his/her patient to our previous study [7].

However, we might have missed some reasons for disagreement between GPs and patients because of the setting of the study. Our recruitment took place solely in a large German city, so that rural areas were not included in our study. Furthermore, our results are only based on multimorbid patients and their GPs in Germany. The problem areas identified in our study might also vary from results of studies in other countries, because of different health care systems.

## Conclusions

Our study showed that the agreement between patients and GPs on complaints and diagnoses is influenced by challenges of disease management in multimorbidity, interface problems between professionals, the patients’ awareness of diseases and their coping with illness. The results illustrate the requirement to enhance the cooperation between GPs, specialists and outpatient care, the demand to improve doctor patient communication and the need for interventions to increase the disease knowledge of the patients.

## Additional files

Additional file 1: **Interview Guide for Focus Groups with GPs.**

Additional file 2: **Interview Guide for Focus Groups with Patients.**
