# Supplementary material for: Reasons for disagreement regarding illnesses between older patients with multimorbidity and their GPs – a qualitative study
Source: BMC Fam Pract. 2015 Jun 2;16:68. doi: 10.1186/s12875-015-0286-x (PMC4450605; doi:10.1186/s12875-015-0286-x)
Supplement: Supplementary file 1 — Interview Guide for Focus Groups with GPs. [file 12875_2015_286_MOESM1_ESM.pdf]

Additional file 1: Interview Guide for Focus Groups with GPs

**Topic**	**Questions/Instructions**
Welcome	1) Welcoming the participants<br>2) Introduction to the topic<br>3) Tips for conduction<br>4) Collecting consent for audio recordings<br>5) Confidentiality reminder<br>6) Personal introductions
Questions regarding the introduction to the topic	When you think of conversations with your multimorbid, elderly patients, are there scenarios where you had the feeling that your patient did not understand everything? Or that the patient couldnôt or didnôt want to listen to you?<br><br>Or do you sometimes have the feeling that patients want to tell you something, but then donôt bring it up?<br><br>Do you have the feeling that your patients donôt understand everything you say? If so, what and why?<br><br>Are there perhaps other reasons why you donôt know certain things about your patients?<br><br>How would you rate the consensus between general practitioner (GP) and patient regarding the patientsó diseases i.e. to what percentage do the patients report a disease which the GP also reports?<br>What would your estimate of the average percentage be?
Introducing the study thus far	A short Power Point presentation on the results of the previous study on the agreement between GPs and patients
Discussing the results	What is your impression of the results on the agreement between GPs and their patients?<br><br>What could be causes for the lacking agreement between GPs and their patients?<br><br>My colleague will write down the topics you name on cards and post them on the wall.<br><br>Possible topics to enquire on:<br>• Treatment through specialists<br>• Certain types of patients<br>• Certain diseases/illnesses<br>• Diagnoses with more intensive therapies vs. non-specific syndromes<br>• Compliance problems<br>• Communication issues<br>• A lack of time<br>• Cognitive problems<br>• é
Enquire on	We have now collected several reasons why GPs and

Topic	Questions/Instructions
Consequences for healthcare provision and recommendations for improvements	patients might not agree on the patientsóillnesses on cards. What does this mean for you?<br><br>What do you recommend to improve healthcare provision?<br><br>+ Questions that arise from the context
Conclusion	1) Final summarization<br>2) Thank you and goodbye
