# Supplementary material for: Reasons for disagreement regarding illnesses between older patients with multimorbidity and their GPs – a qualitative study
Source: BMC Fam Pract. 2015 Jun 2;16:68. doi: 10.1186/s12875-015-0286-x (PMC4450605; doi:10.1186/s12875-015-0286-x)
Supplement: Supplementary file 2 — Interview Guide for Focus Groups with Patients. [file 12875_2015_286_MOESM2_ESM.pdf]

Additional file 2: Interview Guide for Focus Groups with Patients

**Topic**	**Questions/Instructions**
Welcome	1) Welcoming the participants<br>2) Introduction to the topic<br>3) Tips for conduction<br>4) Collecting consent for audio recordings<br>5) Confidentiality reminder<br>6) Personal introductions
Questions regarding the introduction to the topic:	First, I would like to start and open discussion.<br><br>I would like to ask you to think of consultations with your general practitioner (GP)é<br>We all know the feeling, after the consultation, of not having discussed all relevant topics with our GPs adequately, or perhaps not having been able to talk about everything that was important to us.<br><br>Sometimes it was the other way around, and the physician talked so much that it was impossible to remember everything or a few things that the physician said were hard to understand. How is this for you?<br><br>Please tell us about situations where you had the feeling that certain things did not come up that maybe should have been spoken about.<br><br>Are there things which you yourself might not have wanted to report?<br><br>Are there topics which, in your opinion, donôt necessarily need to be discussed with your general practitioner? Rather, perhaps, with a different physician?<br><br>Do you sometimes have the feeling that your GP is not telling you everything about your diseases/illnesses?<br><br>Do you have the feeling that your GP speaks too much medical jargon, so that you cannot understand everything?<br><br>(Which concrete consequences did this have for you?)<br><br>BREAK (3 min)
Introducing the study thus far	A short Power Point presentation on the results of the previous study on the agreement between GPs and patients
Discussing the results	What is your impression of the results on the agreement between GPs and their patients?<br><br>What could be causes for the lacking agreement between GPs and their patients?<br><br>My colleague will write down the topics you name on cards and

Topic	Questions/Instructions
	post them on the wall.<br><br>Possible topics to enquire on:<br>• Treatment through specialists<br>• Certain types of patients<br>• Certain diseases/illnesses<br>• Diagnoses with more intensive therapies vs. non-specific syndromes<br>• Compliance problems<br>• Communication issues<br>• A lack of time<br>• Intimate diseases/illnesses<br>• é
Consequences, desires and any unanswered questions	We have now collected several reasons why GPs and patients might not agree on the patientsóillnesses on cards. What does this mean for you?<br><br>What would you desire that could help improve healthcare provision?<br>e.g. regarding the communication issues problem
Conclusion	1) Final summarization<br>2) Thank you and goodbye
